# Single-port laparoscopy-assisted trans-scrotal hernia sac ligation for pediatric male inguinal hernia

**DOI:** 10.3389/fsurg.2022.944004

**Published:** 2022-11-11

**Authors:** Tian Hang, Qichao Ma, Zhihua Hong, Jianfeng Wang, Zhicai Ling, Houwei Lin

**Affiliations:** ^1^ Department of Pediatric Surgery, Jiaxing Women and Children Hospital Affiliated to Wenzhou Medical University, Jiaxing, China; ^2^ Department of Orthopedics, Shanghai Children’s Hospital, School of Medicine, Shanghai Jiaotong University, Shanghai, China; ^3^ Department of Pediatric Urology, Xinhua Hospital Affiliated to Shanghai Jiaotong University School of Medicine, Shanghai, China

**Keywords:** single-port, laparoscopic trans-scrotal, inguinal hernia, procedure, minimal invasive surgery

## Abstract

**Objective:**

We report the introduction of a novel single-port laparoscopic-assisted trans-scrotal hernia sac ligation (LAT-HSL) technique for the treatment of inguinal hernias in pediatric males. In this article, we describe the LAT-HSL technique and the outcomes.

**Methods:**

Twenty-five male children with confirmed unilateral inguinal hernia who underwent surgical treatment from January 2020 to September 2021 were selected for this study. All children underwent surgical treatment with LAT-HSL, and the operative time, hospital stay, and postoperative results and complications were recorded.

**Results:**

All 25 cases underwent LAT-HSL with minimal perioperative complications, and all children were successfully discharged from the hospital postoperatively. At the postoperative follow-up, there was no retraction or atrophy of the testes, no incisional infection, no chronic pain, no urinary retention, and no recurrent hernias.

**Conclusion:**

Single-port LAT-HSL allows for rapid and accurate localization of the extra-abdominal hernia sac. The method is safe, easy to perform, and adaptable. Additionally, the scar is hidden, and the operation time is short.

## Introduction

Recent reports show that single-port laparoscopic inguinal hernia repair has been widely accepted by pediatric surgeons (1, 2). Compared with traditional open surgery, this technique provides a good field of vision, better cosmetic effect, allows exploration of contralateral patent processus vaginalis hernias, and avoids the anatomy of the inguinal canal. However, single-port laparoscopic hernia repair is sometimes difficult to perform in complicated inguinal hernias, such as sliding hernias. Laparoscopic hernia repair has a higher recurrence rate compared with open hernia repair in children (3). Some tissues between the skin and hernia sac, including the nerves and muscles, may be injured by their inclusion in the suture during the procedure (4–7).

In this study, to address these issues, we report the technique and outcomes of the novel single-port laparoscopic-assisted trans-scrotal hernia sac ligation (LAT-HSL) technique for the treatment of inguinal hernias in pediatric males.

## Materials and methods

Twenty-five pediatric male patients with inguinal hernias operated on from January 2020 to September 2021 were selected and enrolled. The patients had a palpable and reproducible mass in the unilateral groin at the time of examination, clear inguinal ultrasound examination showing the affected hernia sac, and no significant abnormalities in preoperative routine blood and coagulation function tests. Case selection criteria: preoperative ultrasonography clearly showing an inguinal hernia, without other abdominal and inguinal diseases. Exclusion criteria: concurrent hydrocele; contralateral internal ring opening not closed intraoperatively; and other comorbidities requiring simultaneous combined surgery or contraindications to surgery.

We recorded the following patient and surgical characteristics: age, intraoperative confirmation of unilateral inguinal hernia, hernia side, operative time, pneumoperitoneum time, hospital stay, and follow-up time. The postoperative complications in these cases were also recorded.

### LAT-HSL surgical technique

**Figure 1:**
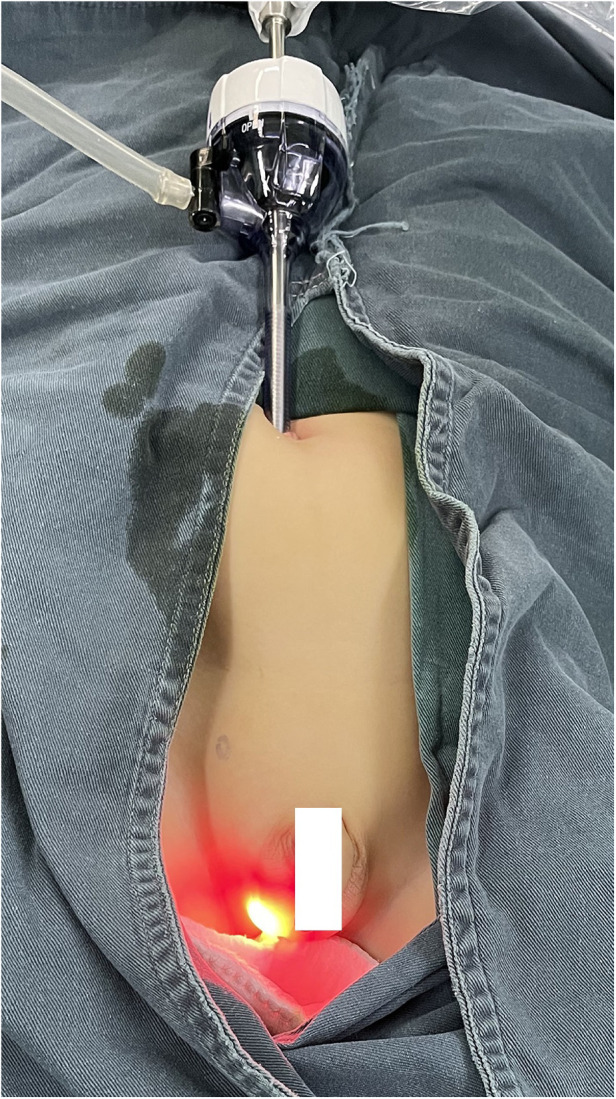
The LAT-HSL technique. Direct laparoscopic view of the hernia sac through the inguinal canal and out the scrotal fold incision. LAT-HSL, laparoscopic-assisted trans-scrotal hernia sac ligation.

**Figure 2:**
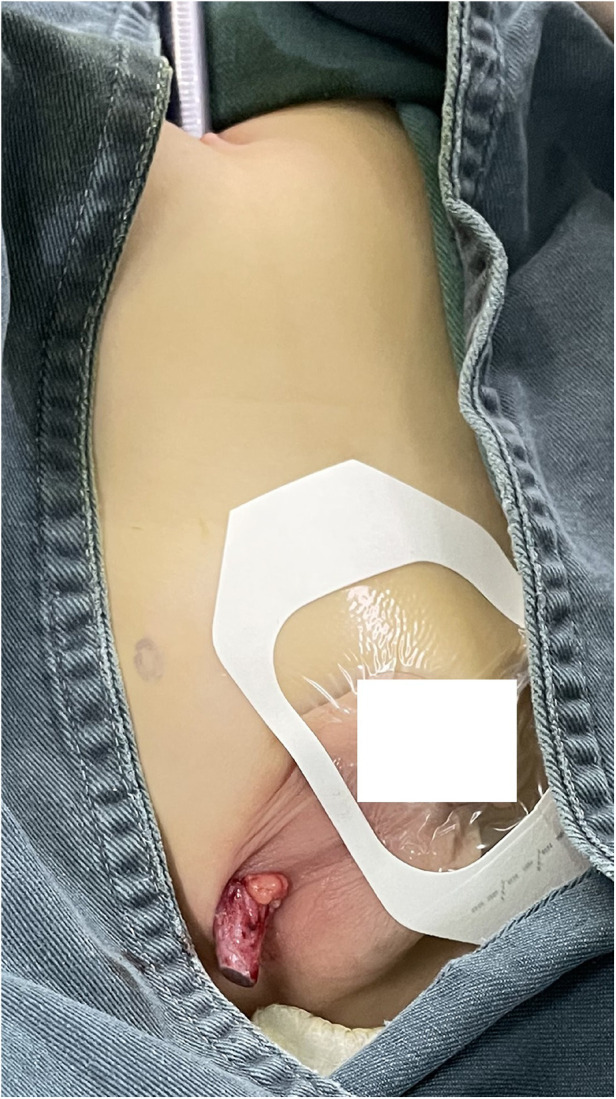
We turn off the light source and release the pneumoperitoneum to keep the hernia sac exposed.

**Figure 3:**
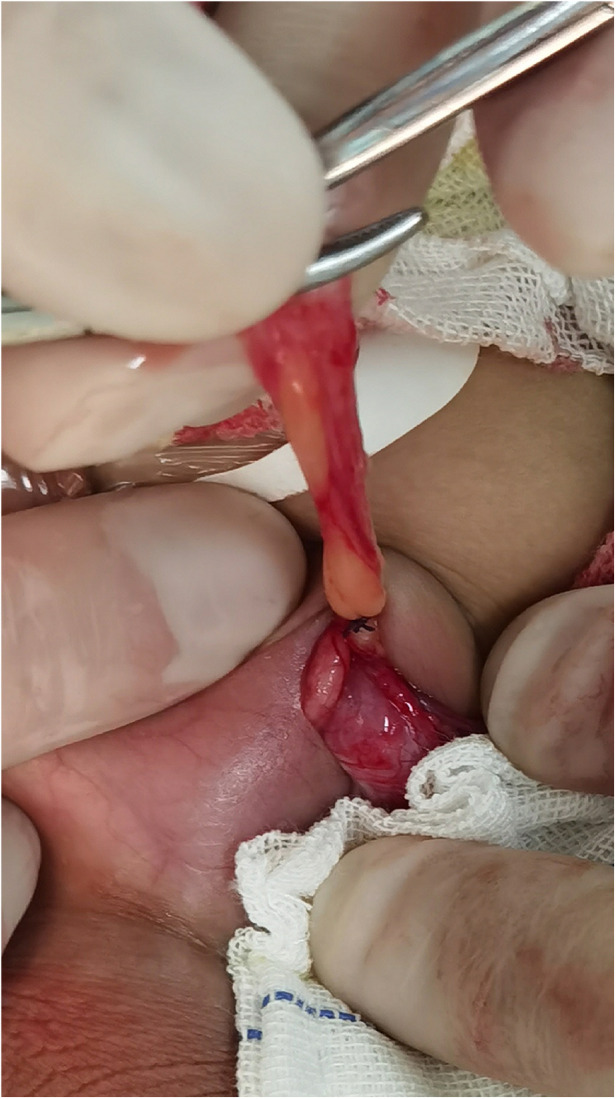
The hernia sac is lifted and elevated to the level of the preperitoneal fat and then ligated.

The procedure was scheduled after informed consent was obtained from the patient's parents, in each case. The following key steps should be noted during LAT-HSL:

1. After induction of general anesthesia, the patient is placed in the supine position. The monitor is placed at the patient's feet. The surgeon stands on the left side of the patient, and the camera assistant stands on the right side. A 5-mm umbilical incision is made, a 5-mm trocar is inserted, and a scrotal incision (5–8 mm in length) is made alongside the scrotum (Figure 1). The surgeon inserts a 5-mm laparoscopic camera through the internal hernia ring into the hernia sac cavity under direct vision, taking care to follow the direction of the inguinal canal (Supplementary Video S1). The surgeon then uses the laparoscope to guide the hernia sac to the scrotal incision and releases the pneumoperitoneum after completing this step.
2. Before recreating the pneumoperitoneum to perform the next step, the camera's light source should be dimmed to avoid thermal burns to the hernia sac cavity and surrounding tissues during the approach (Figure 2).
3. The hernia sac is located and opened through laparoscopic guidance; the tissue around the wall of the hernia sac is bluntly separated to avoid damage to the vas deferens, spermatic vessels, and other important structures; after protecting the spermatic vessels and vas deferens, the hernia sac is lifted and elevated to the level of the preperitoneal fat and then ligated (Figure 3).

**Figure 4:**
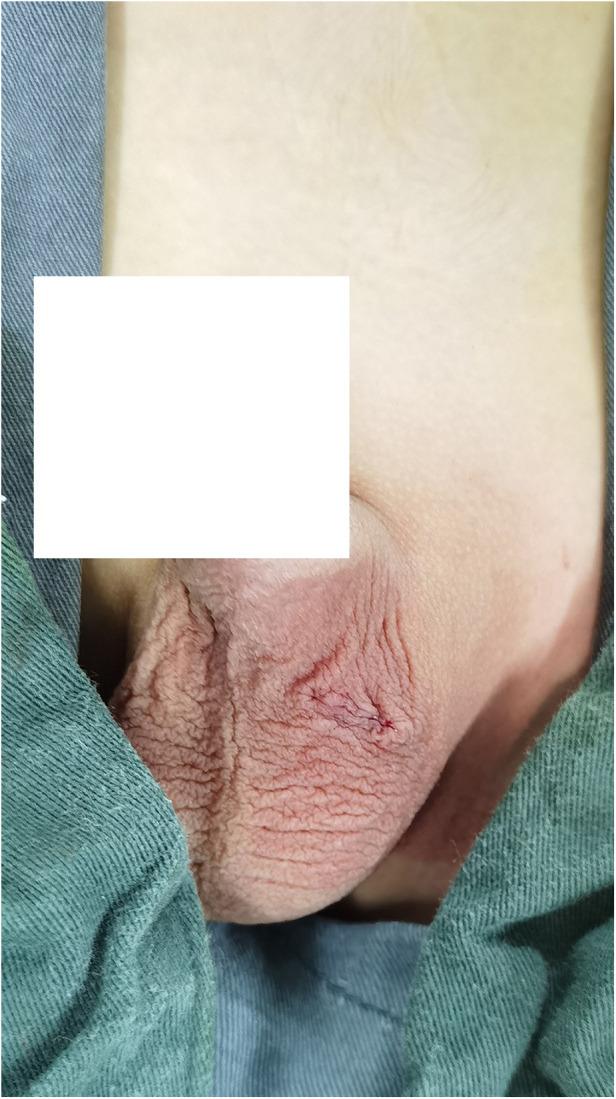
Postoperative scrotal incision appearance.

Pneumoperitoneum is recreated, the morphology of the internal ring opening is observed laparoscopically, and the effect of high ligation is assessed. If the operation is determined to be satisfactory, the incision is closed, the laparoscopic camera is withdrawn, the residual abdominal gas is expelled, and the surgical instruments are inventoried. Then, the umbilical and scrotal incisions are closed intradermally with absorbable suture, and the scrotal incisions are hidden in scrotal folds (Figure 4).

## Results

During the study period, LAT-HSL was performed for the 25 included patients at our institution, all of whom underwent the procedure without incident. The patients’ perioperative characteristics are summarized in Table 1.

**Table 1:** LAT-HSL surgery cases’ characteristic data $(\bar{X}\pm \sigma )$.

Surgical method	LAT-HSL
Number of patients	25
Age: years (range)	(1.19 ± 0.40)
Length of hospital stay, days (range)	(1.92 ± 0.79)
laparoscopic pneumoperitoneum, seconds (range)	(45.25 ± 11.03)
Surgery time, minutes (range)	(20.36 ± 4.33)
Follow-up time, months (range)	(23.12 ± 0.24)
Morbidity	
Swollen scrotum	2 (2/25 = 8%)
Recurrence	0
Hematoma	1 (1/25 = 4%)
Hydrocele	0
Testicular atrophy	0
Chronic pain	0
Urinary retention	0
Wound infection	0

LAT-HSL, laparoscopic-assisted trans-scrotal hernia sac ligation.

Twenty-five male children ranged in age from 9 months to 2 years (mean ± standard deviation age: 1.19 ± 0.40 years). All patients had intraoperative confirmation of unilateral inguinal hernia: 17 hernias were right-sided and 8 were left-sided. The mean operative time was 20.36 ± 4.33 min, mean laparoscopic pneumoperitoneum time was 45.25 ± 11.03 s, mean hospital stay was 1.92 ± 0.79 days, and mean postoperative follow-up time was 23.12 ± 0.24 months.

All patients’ hernias were first hernias, and none had a history of repair. No occult hernias or concurrent hydroceles were identified intraoperatively, and no conversion to open or standard laparoscopic repair was necessary. All cases returned to normal activity before leaving the hospital. There were no serious postoperative complications. Two children presented with scrotal swelling that resolved after conservative observation and reduced activity. One child presented with a scrotal hematoma that eventually resolved spontaneously with sustained local compression therapy. There were no cases of postoperative urinary retention or wound infection. The most recent follow-up was in April 2022. No patients developed chronic pain, secondary hydroceles, or recurrence. The postoperative cosmetic wound results were excellent in all cases.

## Discussion

This new procedure (LAT-HSL) enables the ligation of the hernia sac through a scrotal incision, avoiding dissection of the inguinal canal. The advantages of a traditional scrotal incision for inguinal hernia repair are cosmetic satisfaction and maintaining the integrity of the inguinal canal (8, 9). However, the main shortcomings are difficulty finding the hernia sac and difficulty judging whether the ligation position of the hernia sac is high enough. In open surgery, the hernia sac is pulled upward and then freed completely to the internal inguinal ring where the extraperitoneal fat is found. However, this is an indirect judgment of a high ligation position. If the ligation position is not high enough, it may simply turn a larger hernia sac into a smaller hernia sac. With our method, we can intuitively observe whether the ligation position is high enough, which is also a major advantage of laparoscopic hernia repair. Additionally, in our experience, we have confirmed that a ligation position of the same height as that with laparoscopic hernia repair can be achieved by dissociating the hernia sac to the high position through the scrotal incision. Although some children are less than 1 year old, based on the advantages of this new technology, all children's inner inguinal ring can pass through the 5 mm trocar smoothly.

In previous single-port laparoscopic hernia repair, laparoscopy was used only for observation. In our method, laparoscopy also plays a guiding role. By inserting the laparoscope into the patent processus vaginalis and pushing it into the scrotum, the surgeon can easily find the hernia sac in the scrotal incision. After finding the hernia sac, the sac is lifted and elevated to the level of the preperitoneal fat and ligated.

Previously, we widely used the method of single-port laparoscopic percutaneous extraperitoneal closure for inguinal hernia repair (10). However, with this technique, the inner two-hooked cannula may be an insufficient device for the separation of the spermatic cord, especially in young children. Because of the multiple folds in the hernia sac, it is more difficult to pass the cannula, which may lead to hernia recurrence or the formation of a hydrocele owing to failure of complete ligation of the hernia sac. In some cases, auxiliary forceps need to be added to increase the wound. Additionally, the longer pneumoperitoneum time is a negative factor in the anesthesia management of young children.

Anatomically, in LAT-HSL, the scrotal incision, internal inguinal ring, and umbilicus are not in a straight line. However, owing to the good compliance of children's tissues and muscles, it is quite easy to pass a laparoscope through these three positions, as shown in our Supplementary Video. We turn off the laparoscopic light source after the laparoscope reaches the bottom of the hernia sac to avoid tissue burns from the heat generated by the light source. After the tip of the laparoscope emerges from the scrotal incision, we clamp the bottom of the hernia sac with mosquito forceps and then we release the pneumoperitoneum. After repairing the hernia, we recreate the pneumoperitoneum to confirm the ligation position. This ensures the shortest pneumoperitoneum time and minimizes the impact on the child's respiratory circulation during the operation. With our method, only approximately 45 s of pneumoperitoneum time is required, including the time for the laparoscope to enter the hernia sac and the time to observe the ligation position after ligation. Owing to the short pneumoperitoneum time, low-pressure carbon dioxide pneumoperitoneum (we usually use a pressure of 6–7 mmHg and a flow rate of 2 L/min) will not seriously affect the child's respiration, circulation, or carbon dioxide retention (11).

The scrotal incision is located at the scrotal fold with LAT-HSL; therefore, the incision is concealed and tension-free, and as the appearance is same achieved with general single-port laparoscopy, after healing. There was no significant difference in operation time between our method and that of single-port laparoscopic hernia repair; however, the pneumoperitoneum time was noticeably shorter.

## Conclusions

The LAT-HSL surgical technique is a safe, effective, and esthetically pleasing option for pediatric inguinal hernia repair. Based on the concepts of minimally invasive treatment and concealed scars, LAT-HSL offers a new solution for the minimally invasive treatment of inguinal hernias in male children. Our results showed that this interesting new surgical method is feasible and that no obvious major complications were observed in the short-term follow-up in this small case series. In future research, we will increase our cases and increase control to verify the reliability of this technique.

## Data Availability

The raw data supporting the conclusions of this article will be made available by the authors, without undue reservation.
